# Effects of cerium oxide (CeO_2_) on liver tissue in liver ischemia-reperfusion injury in rats undergoing desflurane anesthesia

**DOI:** 10.1186/s12871-023-01999-0

**Published:** 2023-02-03

**Authors:** Huseyin Gobut, Aysegul Kucuk, Necmiye Şengel, Mustafa Arslan, Cagrı Ozdemir, Tulay Mortas, Esat Kasapbası, Omer Kurtipek, Mustafa Kavutcu

**Affiliations:** 1 Department of General Surgery, Gazi University Faculty of Medicine, Ankara, Turkey; 2 Department of Physiology, Kutahya Health Sciences University Faculty of Medicine, Kutahya, Turkey; 3 Department of Oral and Maxillofacial Surgery (as a specialist in Anesthesiology and Reanimation), Gazi University Faculty of Dentistry, Ankara, Turkey; 4 Department of Anesthesiology and Reanimation, Gazi University Faculty of Medicine, 06510 Ankara, Turkey; 5 Gazi University, Life Sciences and Application Research Centre, Ankara, Turkey; 6 Department of Histology and Embryology, Kırıkkale University Faculty of Medicine, Kırıkkale, Turkey; 7 Department of Medical Biochemistry, Gazi University Faculty of Medicine, Ankara, Turkey

**Keywords:** Cerium oxide, Desflurane, Ischemia-reperfusion, Liver, Biochemical analysis, Histopathological analysis

## Abstract

**Introduction:**

During liver surgery and transplantation, periods of partial or total vascular occlusion are inevitable and result in ischemia-reperfusion injury (IRI). Nanomedicine uses the latest technology, which has emerged with interdisciplinary effects, such as biomedical sciences, physics, and engineering, to protect and improve human health. Interdisciplinary research has brought along the introduction of antioxidant nanoparticles as potential therapeutics. The goal of this study was to investigate the effects of cerium oxide (CeO_2_) administration and desflurane anesthesia on liver tissue in liver IR injury.

**Material and methods:**

Thirty rats were randomly divided into five groups: control (C), ischemia-reperfusion (IR), IR-desflurane (IRD), cerium oxide-ischemia reperfusion (CeO_2_-IR), and cerium oxide-ischemia reperfusion-desflurane (CeO_2_-IRD). In the IR, IRD, and CeO_2_-IRD groups, hepatic ischemia was induced after the porta hepatis was clamped for 120 min, followed by 120 min of reperfusion. Intraperitoneal 0.5 mg/kg CeO_2_ was administered to the CeO_2_ groups 30 min before ischemia. Desflurane (6%) was administered to the IRD and CeO_2_-IRD groups during IR. All groups were sacrificed under anesthesia. Liver tissue samples were examined under a light microscope by staining with hematoxylin-eosin (H&E). Malondialdehyde (MDA) levels, catalase (CAT), glutathione-s-transferase (GST), and arylesterase (ARE) enzyme activities were measured in the tissue samples.

**Results:**

The IR group had considerably more hydropic degeneration, sinusoidal dilatation, and parenchymal mononuclear cell infiltration than the IRD, CeO_2_-IR, and CeO_2_-IRD groups. Catalase and GST enzyme activity were significantly higher in the CeO_2_-IR group than in the IR group. The MDA levels were found to be significantly lower in the IRD, CeO_2_-IR, and CeO_2_-IRD groups than in the IR group.

**Conclusion:**

Intraperitoneal CeO_2_ with desflurane reduced oxidative stress and corrected liver damage.

## Introduction

The Pringle maneuver (flow control) or complete exclusion of vascular structures are used in surgical techniques utilized during excision of intrahepatic lesions or liver transplantation, which causes an ischemia period [1]. Ischemia-reperfusion injury (IRI) resulting from the redirection of blood flow to ischemic tissue can also affect the ischemic organ and distant organs, leading to multiorgan failure [2]. The liver is an organ with a high blood supply, so it is very often exposed to the effects of IRI. Restoration of blood flow after ischemia creates a tendency for injury, which aggravates the damage of ischemia caused by surgery [3].

Ischemia-reperfusion injury is an important cause of liver injury during surgical procedures, such as hepatic resections and liver transplantation [4]. In addition, IRI is a dynamic process involving the release of cytokines and reactive oxygen species (ROS), together with the activation of Kupffer cells and neutrophils, which can result in hepatocyte and sinusoidal endothelial cell death [5].

It is of great importance to study experimental models to examine the mechanisms of hepatic IRI and new surgical and pharmacologic strategies to minimize IRI and to develop a new therapeutic approach to improve liver function after liver surgery and transplantation.

Various agents have been studied to reduce oxidative damage as a result of IRI in various organs. These include volatile anesthetics. In these studies, it was stated that sevoflurane and desflurane had immunomodulatory effects [6, 7], and in a study conducted in rats in which a liver IR model was used, increased aspartate transaminase (AST) and alanine aminotransferase (ALT) values decreased with desflurane administration [8].

Different results are also available in the literature. In a study evaluating the effects of propofol, sevoflurane, and desflurane on oxidative stress, it was reported that MDA levels were higher and that serum and alveolar glutathione peroxidase (GPX) levels were lower in the desflurane-administered group than in the other anesthetic-administered group [9]. Tosun et al. stated that desflurane increased tumor necrosis factor (TNF-α) and nuclear factor kappa B (NF-kB) levels, decreased total glutathione (GSH) levels, and increased polynuclear lymphocyte infiltration, hemorrhage, alveolar damage, and edema [10].

Cerium oxide nanoparticles have antioxidant [11] and anti-inflammatory [12] effects. Reactive oxygen species are an important component of inflammation that can lead to pathogenic consequences by affecting normal cellular activities. In addition, by affecting the fatty acid components of the phospholipid bilayer oxidatively, it can damage the cell membranes; it can also damage DNA structure and proteins [13]. Cerium oxide nanoparticles act as potent antioxidants due to the redox switch of cerium ions in the 3+ and in the 4+ valence state in the oxide depending on the physiological environment [11]. The Ce^4+^ sites are responsible for the oxidation of H_2_O_2_ as catalase (CAT)-mimetics, and the Ce^3+^ sites are known to remove **•**OH via redox reactions and clear O_2_− via superoxide dismutase (SOD)-mimetics [14].

There are also some studies reporting that CeO_2_ administration reduces hepatic IR damage in animals [14, 15].

With this study, we wanted to contribute to the limited literature by examining the combined effects of desflurane and CeO_2_ on hepatic tissue in hepatic IRI in rats.

## Materials and methods

Procedures were approved by the Gazi University Ethical Committee of Experimental Animals (G.Ü.ET-22.032) and conducted in the Gazi University Animal Laboratory, and carried out in accordance with ARRIVE guidelines. All laboratory procedures were in accordance with the Guide for the Care and Use of Laboratory Animals in Ankara, Turkey. In this study, a total of 30 female Wistar albino rats (aged 5-month-old), weighing between 200 and 250 g, were used. Animals were housed under identical environmental conditions and kept at a neutral temperature (20–21 °C) under a 12:12 hour photoperiod. Food and water were available ad libitum.

### Experimental groups

A total of 30 rats were randomly assigned and equally (*n* = 6) divided into the following five groups: control, IR, IRD, CeO_2_ + IR, and CeO_2_ + IRD. All surgical procedures were performed under general anesthesia. An intramuscular injection of 50 mg/kg ketamine hydrochloride (500 mg/10 ml, Ketalar vial, Parke-Davis, Pfizer Inc.) + 10 mg/kg xylazine hydrochloride (Alfazyne vial 2%; Ege Vet, Ltd.) was administered for anesthesia. The procedure was performed under a warming lamp with the rats in the supine position. In all groups, after skin asepsis was achieved, a midline abdominal incision was performed on the rats, and the porta hepatis was then explored. Anesthesia was maintained in the control, CeO_2_, IR, and CeO_2_ + IR groups, which did not receive desflurane, with injections of 20 mg/kg ketamine with 5 mg/kg xylazine if a positive reaction to surgical stress or intermittent tail pinch was observed. Following the end of the reperfusion period, all rats were anesthetized with ketamine (50 mg/kg) and xylazine (10 mg/kg) and sacrificed by collecting blood (5–10 ml) from their abdominal aortas. After the heartbeat and respiration ceased, the rats were monitored for a further 2 min to confirm death. The liver tissue of the rats was excised after sacrifice. Liver tissue samples were collected and kept in 10% formalin solution and serum samples at − 80 °C, and biochemical and histopathological tests were performed. The decision on the CeO_2_ application dose and time was made based on the work done by Ozdemirkan et al. [16].

#### Control group (C group)

A midline laparotomy was the sole surgical procedure, without any additional intervention.

#### Ischemia-reperfusion group (IR group)

A midline laparotomy was performed. For 120 min, an atraumatic micro clamp was placed on the porta hepatis, the clamp was withdrawn, and the liver was reperfused for another 120 min.

#### Ischemia reperfusion-desflurane group (IRD group)

A midline laparotomy was performed. An atraumatic microvascular clamp was placed on the porta hepatis for 120 min, and then the clamp was withdrawn and reperfused for 120 min. During the ischemia period, anesthetic gas vaporizers were calibrated in a transparent plastic box, and desflurane (6%) was administered to the rats by setting the minimum alveolar concentration (MAC) at 1.

#### Cerium oxide-ischemia reperfusion group (CeO_2_-IR group)

Cerium oxide was given (0.5 mg/kg) 30 min before the ischemia period. A midline laparotomy was performed. An atraumatic micro clamp was placed on the porta hepatis for 120 min, the clamp was removed, and the liver was reperfused for another 120 min.

#### Cerium oxide-ischemia reperfusion-desflurane group (CeO_2_-IRD group)

Cerium oxide was administered (0.5 mg/kg) intraperitoneally 30 min before the ischemia period. A midline laparotomy was performed. An atraumatic microvascular clamp was placed on the porta hepatis for 120 min; then, the clamp was removed and the liver was reperfused for another 120 min. During the ischemia period, desflurane was administered with a 6% inspiratory concentration in a transparent plastic box.

### Histopathological evaluation

The histopathological assessment was performed in the Department of Histology at Kırıkkale University. After the fixation process, the specimens were prepared with paraffin blocks. Tissue sections of 5 μm were stained using hematoxylin and eosin (H&E). Histopathological assessments and scoring were performed under light microscopy. The same pathologist performed the histologic evaluations in a blinded manner.

In the histopathological examination, each preparation was examined for hepatocyte degeneration, sinusoidal dilatation, prenecrotic cells, and mononuclear (MN) cellular infiltration in the parenchyma. The semiquantitative evaluation technique used by Abdel-Wahhab et al. [17] was used to interpret the structural changes investigated in the hepatic tissues of the control and research groups. Accordingly, (−) (negative points) represents no structural changes; (+) (one positive point): mild changes; (++) (two positive points): medium changes; and (+++) (three positive points): severe structural changes.

### Biochemical evaluation

The biochemical examination was conducted in the Department of Medical Biochemistry at Gazi University. Oxidative stress and lipid peroxidation in liver tissue were evaluated by measuring thiobarbituric acid reactive substance (TBARS) levels and CAT and GST enzyme activities.

The TBARS assay was performed to determine lipid peroxidation using the thiobarbituric acid method [18]. The TBARS measurements were conducted based on the reaction of MDA with thiobarbituric acid (TBA), which forms a pink pigment with an absorption maximum at 532 nm in acidic pH, and 1,1,3,3-tetraethoxypropane was used as a standard MDA solution.

The CAT activity was based on the measurement of absorbance decrease due to H2O2 consumption at 240 nm, as described in the Aebi method [19].

The GST enzyme activity was measured using the method described by Habig et al. [20]. The GST activity method is based on the measurement of absorbance increase at 340 nm due to the reduction of dinitrophenyl glutathione (DNPG). The results are expressed in international units per milligram of protein.

The ARE activity was measured at the rate of hydrolysis of phenyl acetate by monitoring the change in absorbance at 270 nm and at 25 °C using Brites’s method [21].

### Statistical analysis

The Statistical Package for the Social Sciences (SPSS, Chicago, IL, USA) 20.0 for Windows was used for the statistical analysis. Each categorical variable was analyzed using the Shapiro-Wilk test. Biochemical and histopathological parameters were tested using the Kruskal-Wallis test, Bonferroni correction test, and Mann-Whitney U test. A statistical value of less than 0.05 was considered significant. All values are expressed as the mean ± standard error (mean ± SE).

## Results

### Histopathological results

Hydropic degeneration, sinusoidal dilation, pycnotic nuclei, and parenchymal mononuclear cell infiltration were found to be significantly different between the groups (*p* = 0.006, *p* = 0.033, *p* = 0.027, and *p* = 0.042, respectively).

**Fig. 1:**
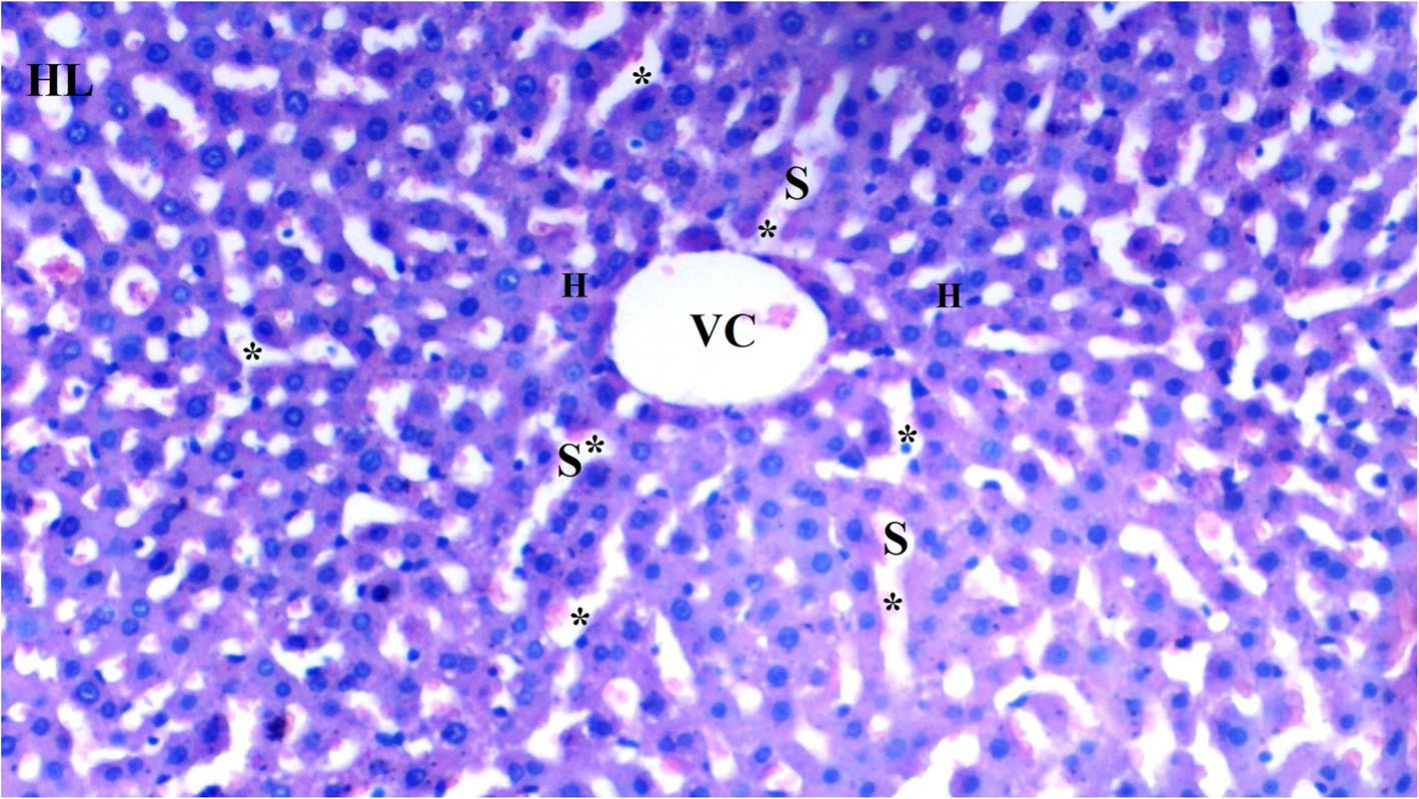
Light microscopic view of hepatic tissue of group C; normal liver tissue (HL: hepatic lobules; VC: vena centralis; *: hepatocyte; S*:Sinusoids; H:Hepatocyte)

**Fig. 2:**
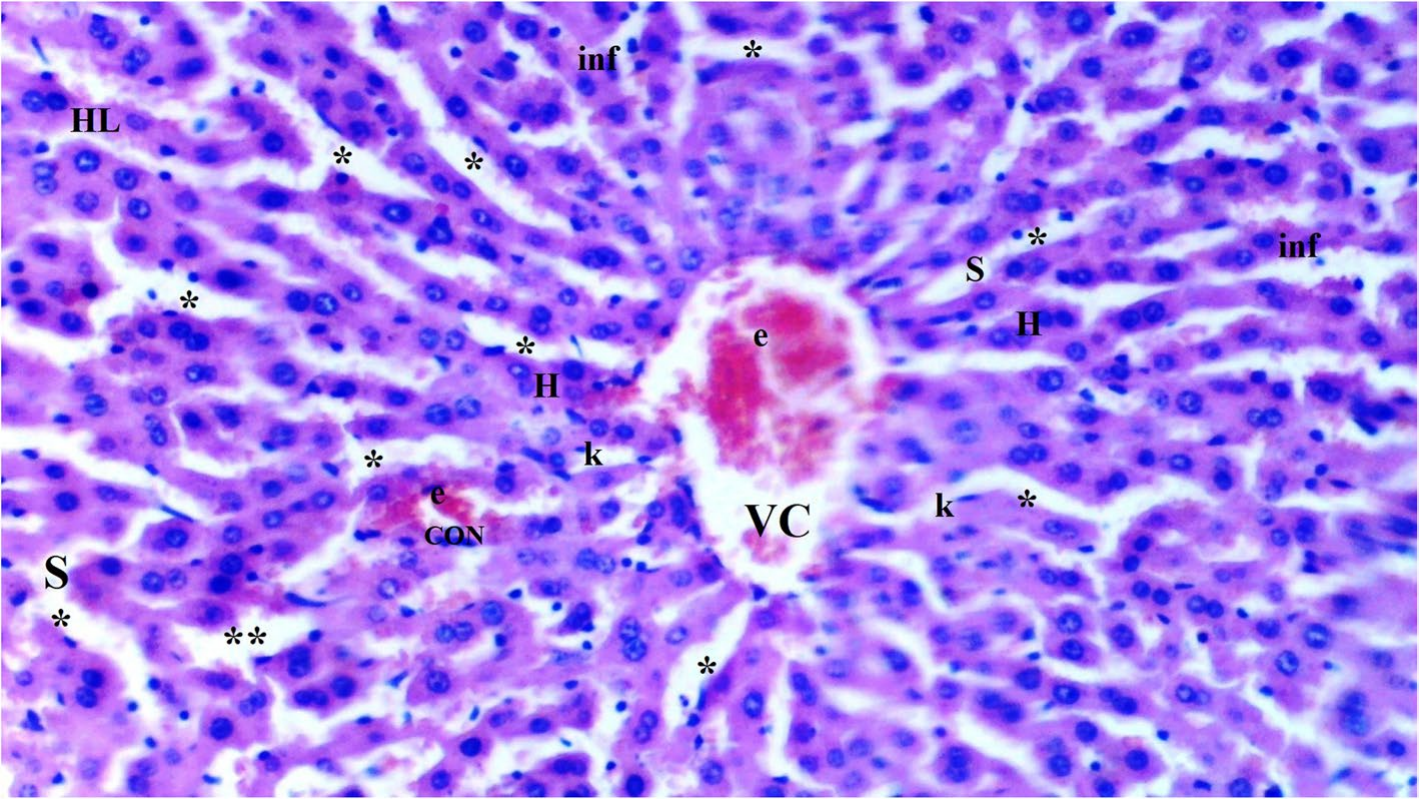
Light microscopic view of hepatic tissue of group IR (HL: hepatic lobules; VC: vena centralis; e: erythrocyte; CON: congestion; **: sinusoids; k: Kupffer cell hyperplasia; *: hepatocytes; inf: inflammation; S:Sinusoid; H:Hepatocyte)

**Fig. 3:**
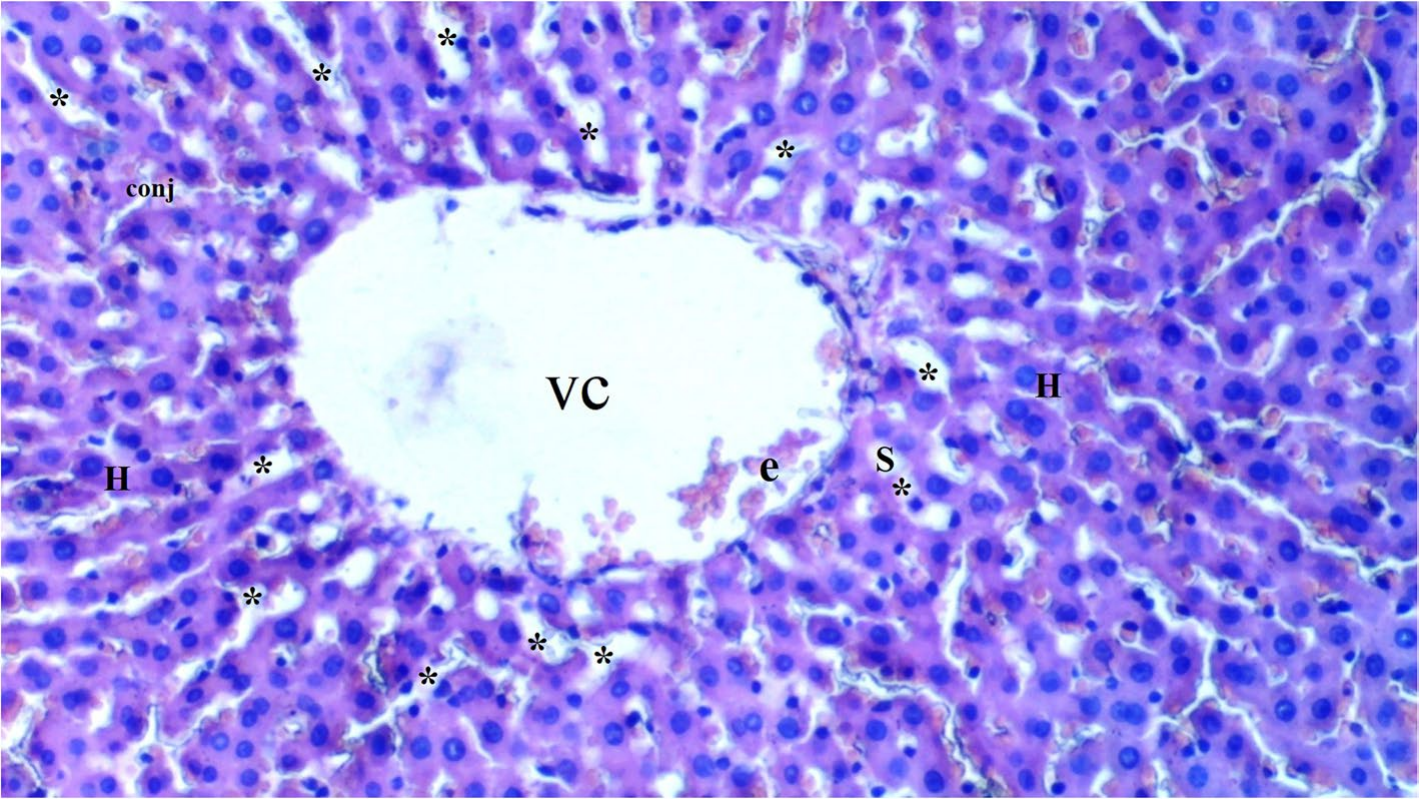
Light microscopic view of hepatic tissue of the CeO2-IR group (VC: vena centralis; conj: congestion; *: hepatocytes; e: erythrocyte; S:Sinusoid; H:Hepotocyte)

**Fig. 4:**
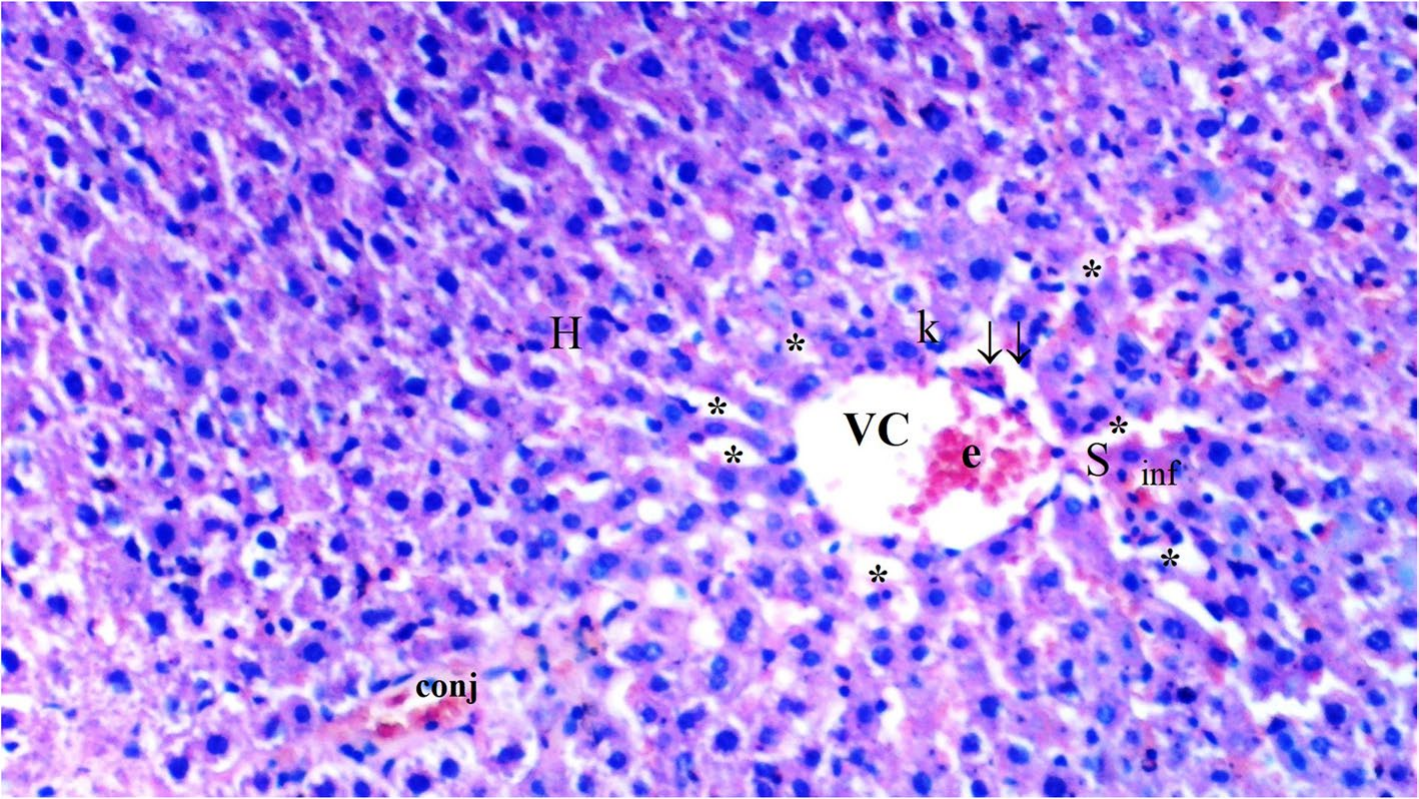
Light microscopic view of hepatic tissue of group IRD (VC: vena centralis; e: erythrocyte; conj: congestion; inf: inflammation; *: hepatocytes; k: Kupffer cell hyperplasia; ↓↓: infiltration; S:Sinusoid; H:Hepatocyte)

**Fig. 5:**
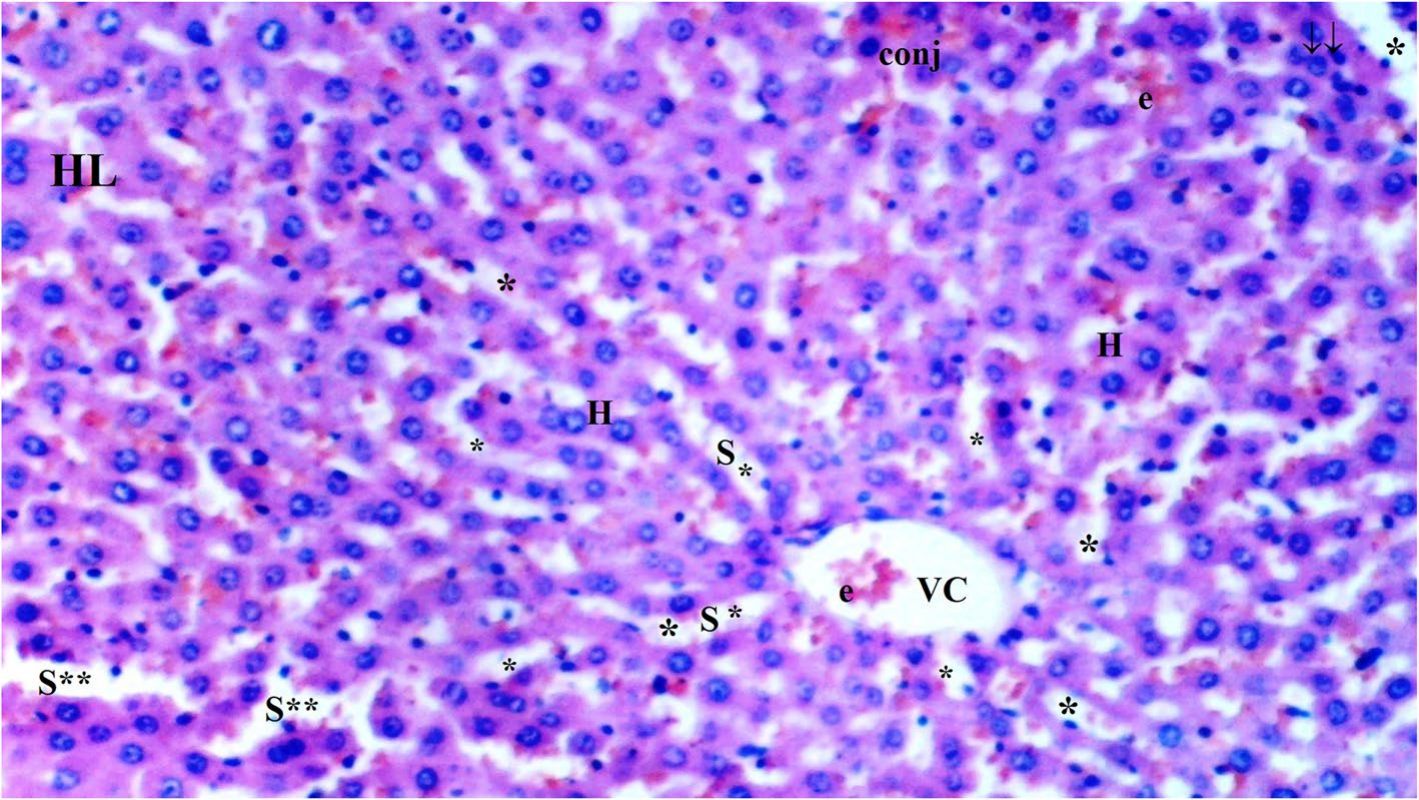
Light microscopic view of hepatic tissue of group CeO2-IRD (HL: hepatic lobules; VC: vena centralis; e: erythrocyte; conj: congestion; **: sinusoids; ↓↓: infiltration; *: hepatocytes; S:Sinusoid; H:Hepatocyte)

Hydropic degeneration was more common in the IR group than in the control group (*p* = 0.001). Hydropic degeneration was found to be significantly lower in the CeO_2_-IR, IRD, and CeO_2_-IRD groups than in the IR group (*p* = 0.002, *p* = 0.032, and *p* = 0.009, respectively) (Table 1, Figs.1, 2, 3, 4, and 5).

**Table 1:** Histopathological Data of Liver Tissue [Mean ± SE]

	Group C (*n =* 6)	Group IR (*n =* 6)	Group CeO2-IR (*n =* 6)	Group IRD (*n =* 6)	Group CeO2-IRD (*n =* 6)	P**
Hydropic Degeneration	0.33 ± 0.21*	1.50 ± 0.22	0.50 ± 0.22*	0.83 ± 0.17*	0.67 ± 0.21*	0.006
Sinusoidal Dilation	0.33 ± 0.21*	1.67 ± 0.33	0.67 ± 0.21*	0.83 ± 0.31*	0.83 ± 0.31*	0.033
Pycnotic nuclei	0.17 ± 0.17*	1.17 ± 0.17	0.33 ± 0.21*	0.83 ± 0.31+	0.50 ± 0.22*	0.027
Necrosis	0.33 ± 0.21	1.17 ± 0.17	0.50 ± 0.22	0.83 ± 0.31	0.67 ± 0.21	0.130
Parenchymal mononuclear cell infiltration	0.33 ± 0.21*	1.50 ± 0.34	0.50 ± 0.22*	0.83 ± 0.31	0.67 ± 0.21*	0.042

p**: Significance level with Kruskal-Wallis test *p <* 0.05

+*p <* 0.05: Compared with group C

*p < 0.05: Compared with the IR group

Sinusoidal dilatation was more common in the IR group than in the control group (*p* = 0.002). Sinusoidal dilatation was found to be significantly lower in the CeO_2_-IR, IRD, and CeO_2_-IRD groups than in the IR group (*p* = 0.018, *p* = 0.045, and *p* = 0.045, respectively) (Table 2, Figs. 1, 2, 3, 4, and 5).

**Table 2:** Biochemical data of liver tissue [Mean ± SE]

	Group C (*n =* 6)	Group IR (*n =* 6)	Group CeO2-IR (*n =* 6)	Group IRD (*n =* 6)	Group CeO2-IRD (*n =* 6)	P**
MDA (nmol/mg.pro)	0.12 ± 0.01*	0.23 ± 0.01	0.14 ± 0.02*	0.19 ± 0.01+	0.15 ± 0.01*	< 0.0001
CAT (IU/mg.pro)	357.98 ± 43.80*	186.05 ± 15.20	306.17 ± 19.93*	240.15 ± 20.53+	294.58 ± 24.12*	0.001
GST (IU/mg.pro)	1.05 ± 0.06*	0.61 ± 0.04	0.91 ± 0.11*	0.74 ± 0.06+	0.88 ± 0.05*	0.002
ARE (IU/mg.pro)	1.21 ± 0.08*	0.82 ± 0.05	1.03 ± 0.06*	0.98 ± 0.05	1.14 ± 0.10*	0.006

p**: Significance level with Kruskal-Wallis test *p <* 0.05

+*p <* 0.05: Compared with group C

*p < 0.05: Compared with the IR group

Pycnotic nuclei were more common in the IR and IRD groups than in the control group (*p* = 0.004 and *p* = 0.043, respectively). Pycnotic nuclei were found to be significantly lower in the CeO_2_-IR and CeO_2_-IRD groups than in the IR group (*p* = 0.013 and *p* = 0.043, respectively). Pycnotic nuclei were found to be similar between the IR and IRD groups (*p* = 0.297) (Table 1, Figs. 1, 2, 3, 4, and 5).

Parenchymal mononuclear cell infiltration was more common in the IR group than in the control group (*p* = 0.005). Parenchymal mononuclear cell infiltration was significantly lower in the CeO_2_-IR and CeO_2_-IRD groups than in the IR group (*p* = 0.013 and *p* = 0.035, respectively). Parenchymal mononuclear cell infiltration was found to be similar between the IR and IRD groups (*p* = 0.087) (Table 1, Figs. 1, 2, 3, 4, and 5).

Necrosis was found to be similar between the groups (*p* = 0.130) (Table 1, Figs. 1, 2, 3, 4, and 5).

### Biochemical results

**Fig. 6:**
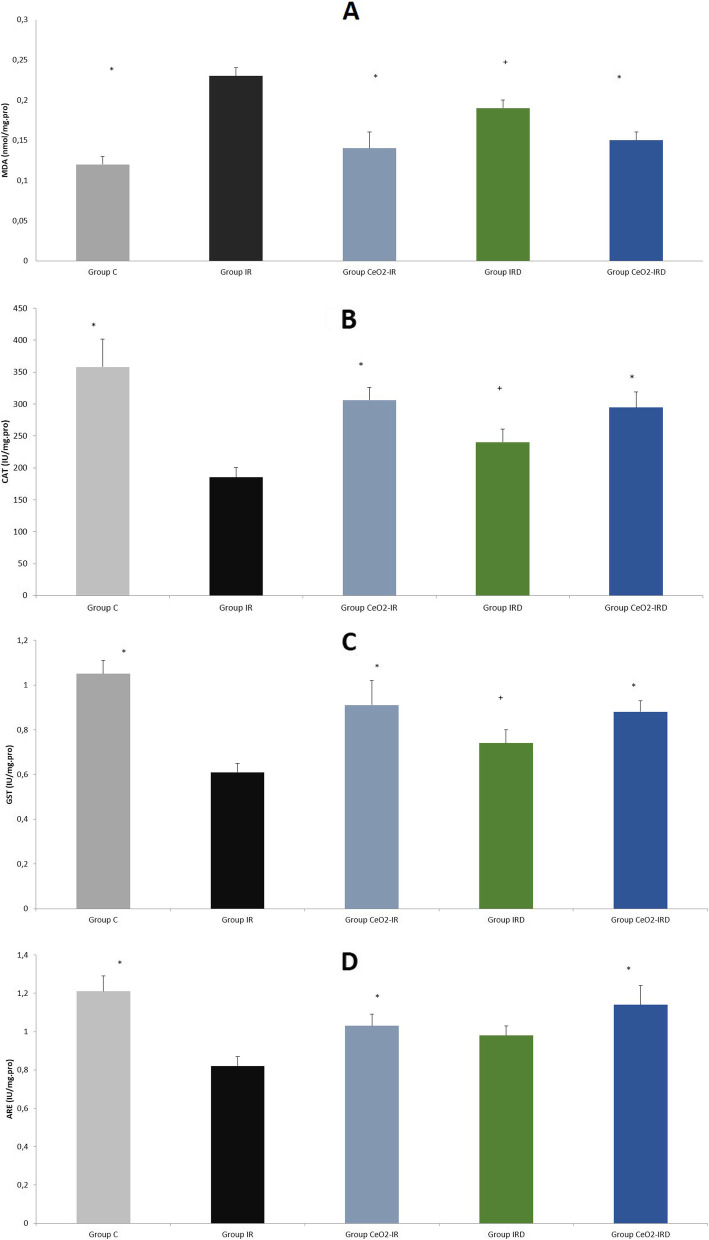
(**A**-**D**): MDA level, CAT, GST, and ARE enzyme activities of liver tissue [Mean ± SE] . +*p <* 0.05: Compared with group C; *p < 0.05: Compared with the IR group

When the levels of MDA in liver tissue were compared between the groups, there was a significant difference (*p* < 0.001). The MDA levels in the IR and IRD groups were found to be considerably higher than those in the control group (*p* < 0.001 and *p* = 0.004, respectively). In addition, the MDA levels were found to be significantly lower in the CeO_2_-IR and CeO_2_-IRD groups than in the IR group (*p* < 0.001 and *p* = 0.001, respectively) (Table 2, Fig. 6A).

In terms of CAT enzyme activity in liver tissue, there was a substantial difference between the groups (*p* = 0.001). Furthermore, the CAT enzyme activity was found to be significantly decreased in the IR and IRD groups compared with the control group (*p* < 0.001 and *p* = 0.004, respectively). The CAT enzyme activities in the CeO_2_-IR and CeO_2_-IRD groups were significantly increased compared to the IR group (*p* = 0.004 and *p* = 0.008, respectively). The CAT enzyme activity was similar between the IR and IRD groups (*p* = 0.164) (Table 2, Fig. 6B).

In terms of GST enzyme activity in liver tissue, there was a substantial difference between the groups (*p* = 0.002). The GST enzyme activity in the IR and IRD groups was considered to be significantly lower than that in the control group (*p* < 0.001 and *p* = 0.004, respectively). The GST enzyme activity was observed to be considerably higher in the IR group than in the CeO_2_-IR and CeO_2_-IRD groups (*p* = 0.005 and *p* = 0.010, respectively). The IR and IRD groups were similar (*p* = 0.188) (Table 2, Fig. 6C).

In terms of ARE enzyme activity in liver tissue, there was a substantial difference between the groups (*p* = 0.006), with ARE enzyme activity that was found to be significantly decreased in the IR group compared with the control group (*p* = 0.001). In the CeO_2_-IR and CeO_2_-IRD groups, ARE enzyme activities were significantly increased compared with those in the IR group (*p* = 0.041 and *p* = 0.003, respectively). The ARE enzyme activity was found to be similar between the IR and IRD groups (*p* = 0.101) (Table 2, Fig. 6D).

## Discussion

In this study, we evaluated the effects of CeO_2_ administration and desflurane anesthesia on liver tissue in liver IR injury. There are few data in the literature regarding the effects of CeO_2_ administration and desflurane anesthesia on hepatic IRI. To investigate the effects of CeO_2_ and CeO_2_ with desflurane on damage, we examined both histopathological and oxidative damage parameters in the liver in IRI. At the end of our study, it was shown that the use of intraperitoneal CeO_2_ and CeO_2_ with desflurane in the liver IR injury model significantly reduced both histopathological damage and oxidative damage in the liver.

The liver is highly dependent on its oxygen supply and is susceptible to hypoxic or anoxic conditions [22]. Hepatic IR injury causes impairment or failure of liver functions, usually following liver resection or transplantation surgery. Hepatic IRI includes both warm and cold IRI—two types that share similar pathophysiological processes. The factors or pathways that have been implicated in the hepatic IRI process include anaerobic metabolism, mitochondria, oxidative stress, intracellular calcium overload, liver Kupffer cells and neutrophils, and cytokines and chemokines [23]. Reactive oxygen species such as hydrogen peroxide (H_2_O_2_), superoxide anion (O_2_−), and hydroxyl radical (HO−) play an important role in reperfusion injury, which causes peroxidation of DNA, proteins, and lipids and several pathologies, including cell death, inflammation, and failure of liver functions [24]. Accordingly, it is important to develop methodologies that can eliminate ROS during reperfusion to prevent and treat hepatic IRI. For this purpose, the potential antioxidant therapeutic agent CeO_2_ has been the subject of experimental studies.

Cerium oxide provides a reduction in ROS by scavenging hydroxyl radicals by activating SOD and CAT [25, 26]. Inorganic nanoparticles can emerge as drug carriers as robust, versatile scaffolds that can adjust nonconjugated biomolecule activities and biological antennas that can be excited in transparent media to develop imaging and therapeutic agents for the early detection and treatment of diseases. Due to their unique physicochemical signatures, they can be easily detected and monitored in physiological environments [27]. Cerium oxide is a nanoparticle that modulates oxidative stress in diseases such as smoking-related oxidative stress, hepatotoxicity, neurodegenerative diseases, retinopathy, cardiomyopathy, obesity, and intestinal IR injury [28, 29].

Desflurane is a safe and effective anesthetic agent that is frequently used in clinics because it provides rapid recovery and rapid extubation [30, 31]. In their study on patients who underwent laparoscopic cholecystectomy, Köksal et al. reported that there was a significant increase in serum MDA levels in the desflurane and sevoflurane groups, and this increase was higher in the desflurane group. They also reported that there was a significant increase in bronchoalveolar lavage MDA levels in the desflurane group and a small but significant increase in serum SOD enzyme activities in the desflurane group and that desflurane might cause more systemic and regional lipid peroxidation than sevoflurane [32, 33]. In another study conducted on patients undergoing laparoscopic abdominal surgery, it was stated that sevoflurane and desflurane showed a protective effect against the increase in MDA and protein carbonyl levels in the perioperative period [33]. When Mangus et al. examined 1291 patients who underwent a liver transplantation, they found that the peak ALT level was the lowest for the desflurane group in the first 7 days after transplantation, followed by the sevoflurane and isoflurane groups. All groups had similar ALT and total bilirubin levels 7 days after transplantation. The 1-year survival rate was similar for all three groups. The authors stated that desflurane or sevoflurane had early hepatic protective effects against IRI, and their long-term outcomes were similar [34].

For these reasons, we wanted to include CeO_2_ in our study with the aim of contributing to the literature on the antioxidant effects of desflurane with its widespread use in the clinic. We preferred to administer CeO_2_ intraperitoneally due to its advantages, such as ease of administration and rapid absorption. We decided on the doses by using previous studies as a guide [35, 36]. In our study, we evaluated oxidative stress and lipid peroxidation in liver tissue by measuring MDA levels, CAT, GST, and ARE enzyme activities. Because blood samples will show general damage, we wanted to study biochemical markers in tissues to show tissue damage.

Malondialdehyde is an important product of lipid peroxidation, and its increase in serum is an indicator of oxidative stress. Glutathione is an important thiol anti-oxidant [36]. Catalase is an important antioxidant enzyme in liver tissue [37]. Paraoxonase-1 (PON-1) is an enzyme synthesized in the liver residing on high-density lipoprotein (HDL) that catalyzes the hydrolysis of various aromatic carboxylic acid esters, organophosphates, and lactones [38]. Paraoxonase-1 has many enzymatic activities, such as lactonase, thiolactonase, arylesterase, and aryldialkylphosphatase (commonly known as paraoxonase) [39]. In humans, a strong negative correlation between PON-1 activity and serum concentrations of lipid oxidation markers has been observed [40].

In our study, hydropic degeneration and sinusoidal dilatation were significantly lower in the CeO_2_-IR, IRD, and CeO_2_-IRD groups than in the IR group. In the IRD group, pycnotic nuclei and parenchymal mononuclear cell infiltration were less than in the IR group, but there was no significant difference. There was no difference between the CeO_2_-IR, CeO_2_-IRD, and IR groups. Necrosis was found to be similar between the groups, but it was found to be less common in the IRD, CeO_2_-IR, and CeO_2_-IRD groups than in the IR group. The MDA levels were found to be significantly lower in the CeO_2_-IR and CeO_2_-IRD groups than in the IR group. The CAT, GST, and ARE enzyme activities in the CeO_2_-IR and CeO_2_-IRD groups were significantly higher than in the IR group. Although there was a significant increase in MDA levels in both the IRD and IR groups compared with the C group, the increase was less in the IRD group; however, there was no significant difference between the IRD and IR groups. The CAT, GST, and ARE enzyme activities were also increased more than in the IR group, but the difference was not significant.

Although desflurane had positive effects in terms of histopathological and oxidative stress, there was no significant difference compared with the IR group. Our finding that intraperitoneally administered CeO_2_ can reduce oxidative stress and liver damage caused by IRI is clear. Although desflurane alone could not provide protective effects similar to those obtained with CeO_2_, it was observed that desflurane, together with CeO_2_, resulted in similar reductions in oxidative stress and injury to the liver.

There are studies with similar results to ours indicating that CeO_2_ has protective effects against IRI. When Manne et al. investigated the hepatic protective effect of CeO_2_ in experimental hepatic IR injury, they observed that histopathological results, such as hepatocyte swelling, vacuolar degeneration, and areas of necrosis in the liver tissues of rats treated with CeO2, improved, and the severity of inflammation decreased. Their findings supported the hepatoprotective effects of CeO_2_ in hepatic IRI [15]. Ozdemirkan et al. investigated lung injury induced by CeO_2_ and desflurane in rats with lower extremity IRI. They stated that serum MDA and nitric oxide levels were lower in the groups treated with CeO_2_, and CeO_2_ together with desflurane, compared with the IR group, and histopathological results were better in these two groups [16].

In conclusion, CeO_2_ administered intraperitoneally at a dose of 0.5 mg/kg 30 min before ischemia alone and in the desflurane-administered group reduced oxidative stress and corrected the liver damage caused by IRI. We think that CeO_2_ administration before IRI and desflurane anesthesia during IRI can have a liver-protective effect in rats. These findings suggest an organ-protective effect of CeO_2_ during desflurane anesthesia. The protective effects of CeO_2_ in different organs under desflurane anesthesia will also be the subject of future studies. It is of great importance that preventive treatments for IRI be implemented in clinics. Nanoparticles are an important topic pending discovery in this field.

## Conclusion

Although desflurane alone could not provide protective effects similar to those obtained with CeO_2_ in hepatic IR injury, it was observed that, together with CeO_2_, it resulted in a similar reduction in oxidative stress and improvement in histopathological results with CeO_2_. These findings implicate an organ-protective effect of CeO_2_ during desflurane anesthesia, which will also offer a foundation for future studies to treat other IRI caused by surgery or liver diseases with therapeutic nanomaterials.

### Limitations

There are some limitations to our study. One was that the rats were anesthetized with ketamine and xylazine. The effects of these agents on oxidative damage could not be excluded. In addition, some parameters could be studied biochemically, and anti-inflammatory cytokine levels could not be studied. It is important to study molecular histopathology to fully understand these mechanisms.

## Data Availability

The data used and analyzed during the current study are available from the corresponding author on reasonable request.
